# Oliver Wendell Holmes

**Published:** 1887-10-22

**Authors:** 


### Oliver Wendell Holmes.

By Dr. Caterina.

(Concluded from p. 24).

But the appreciation of the meaning of these two terms, combined with some slight study of moral teratology, helps greatly to a more charitable view of our fellow-men, with their sins and weaknesses. We know how, if not yet why, they exist. "Suppose," says Dr. Holmes, "that one should go to the worst quarter of the city and pick out the worst-looking child of the worst couple he could find, and then train him up successively at the School for Infant Rogues, the Academy for Young Scamps, and the College for Complete Criminal Education, would it be reasonable to expect a François Xavier or a Henry Martyn to be the result of such a training? I have seen men and women so disinterested and noble and devoted to the best works, that it appeared to me if any good and faithful servant was entitled to enter into the joys of his Lord, such as these might be. But I do not know that I ever met with a human being who seemed to me to have a stronger claim on the pitying consideration and kindness of his Maker than a wretched, puny, crippled, stunted child, that I saw in Newgate, who was pointed out to me as one of the most notorious and inveterate little thieves in London. I have no doubt that some of those who were looking at this pitiable morbid secretion of the diseased social organism thought they were very virtuous for hating him so heartily."

This power of seeing the bearing of scientific principles on moral and intellectual questions, sometimes considered beyond the domain of science, gives a special charm to Dr. Holmes's work, and perhaps constitutes its greatest value. He is no materialist, as the word is generally understood, and he protests against all the gems of thought and the flowers of fancy being crushed by the brick and mortar of mere facts. "All generous minds have a horror of what are commonly called 'facts'," he says. "They are the brute beasts of the intellectual domains." And it is not impossible that if one should venture to check one of his impassioned utterances by a suggestion that facts did not bear out his inferences, he would reply in the immortal words of his fellow-countryman, Mr. Barnum, "So much the wusser for the facts." Indeed, he expresses that very sentiment in language somewhat less terse and laconic: "If I had not force enough to project a principle full in the face of the half-dozen most obvious facts which seem to contradict it, I would think only in single file from this day forward. Scientific knowledge, even in the most modest persons, has mingled with it a something which partakes of insolence."

But scientific men themselves will tell you that a principle is greater than the facts from which it is deduced; it has an elasticity which they have not; although, as Herbert Spencer points out, facts which are marshalled to confute a theory often, when looked at more closely, serve to support it, and have to join the ranks of the enemy. And while Dr. Holmes has a certain contempt for those who pin their faith to a small collection of opinions or observations—mere fossil facts, with all their living, nutritive, productive life turned to stone long ago—he accepts gladly that doctrine of "eternal hope" which we call the Principle of Evolution. "Do we not all hope, at least," he asks, "that the doctrine of man's being a blighted abortion, a miserable disappointment to his Creator, and hostile and hateful to Him from his birth, may give way to the belief that he is the latest terrestrial manifestation of an ever-upward striving movement of divine power? If there lives a man who does not want to disbelieve the popular notions about the condition and destiny of the bulk of his race, I should like to have him look me in the face and tell me so."

There is, however, one point on which I, and I think most medical men, will disagree with Dr. Holmes—that is, if we are to take the sentiments expressed by the Master of Arts as representing his. The Master complains that the only disadvantage which comes of the improved sanitary conditions of modern times is, "that the little good-for-nothings that come of utterly used-up and worn-out stock, and ought to die, can't die, to save their lives! So they grow up, to dilute the vigour of the race with skim-milk vitality. The little sham organisations — the worm-eaten windfalls, for that's what they look like—hang on to the boughs of life like 'froze-'n'-thaws.'"

This is a protest that has often been made, and sometimes even brought as something very like an accusation against doctors—that the quantity of cure of disease and preservation of life they may take credit for is large, but that the quality is not always first-rate. There are some people, say the exacting critics, who, because of some lack of quality in mind or body, aren't worth keeping alive. God knows best, we answer; but though that reply is in some measure conclusive, we may at least suggest a reason why these apparently valueless lives are worth preserving. Thews and sinews are not everything; we want other things as well as physical strength nowadays; and the man who cannot bequeath strong limbs and a vigorous organisation to his children, may enrich the race at large with new ideas or inventions which mankind could ill do without. The Spartan method of bringing up children does not commend itself to the practical intelligence of the modern mind any more than to its moral sentiments. We are more far-sighted than our ancestors, and can believe, while as yet we see not how, that organisms which seem useless to humanity under existing conditions, may be of the highest value in some future phase of human development. We cannot yet tell how much we or our children may owe to some of the "froze-'n'-thaws."

But this is a difference merely in the point of view. One cannot be at both ends of a line at the same moment, either mentally or bodily; and if we concede that there are some lives—sad, painstricken, hopeless, or sinful lives—whose *raison d'être* is a mystery to us, we may be quite sure that the Doctor will permit us to enjoy the rolling verses of *Paradise Lost*, though the author of them was blind; and will share our delight in that *Ode to the Nightingale*, with which one John Keats, a surgeon's apprentice of a phthisical constitution, has enriched the English tongue.

Nor would one quarrel over a point like this with a writer who is perhaps the most eminently companionable author now living. Take up his works in any mood you please, and it will be strange if he will not give you something to suit it. He will laugh with you, and lavish upon you epigrams which you would like to pass off as your own, if it were not so certain that the majority of your acquaintance know them as well as you, and know too who first uttered them. If you are serious he will preach you a very good sermon, or give you a lecture on some scientific subject; or talk to you of trees and the scent of flowers; or discourse to you of antiquities or literature; or perhaps read you some poem of his own, fanciful in its tenderness or humour, and finely perfect in its workmanship.

All his poetry is not of the highest order. He does not take his muse seriously enough; for though Pegasus in harness may be a sorry sight, he is more likely to be kept in good working condition that way than when he is only occasionally called on for an after-dinner canter—an exercise which nobody likes to be too severe, and in which the steed's paces are not too closely criticised. But the man who wrote "Under the Violets" is a poet who cannot be disqualified in the race for the laurel crown. And the gift of humour which he possesses is altogether precious in an age which, among all its virtues, is not greatly marked by a cheerful spirit. There are, it is true, some among us who have reached such a point of sensitiveness that the very name of a comic poem sets them a-shuddering ; but even they could hardly complain of that touch of humour which gives a subtler point to the pathos of " The Old Man's Dream," or " The Boys"; and if they don't appreciate the poem on " Contentment" we can only be sorry for the lack of taste they display. If, as has been said, the essence of wit consists in a certain surprise given to the intellect, one cannot but admit the wit of a poem on contentment which contains such a stanza as this—

> " Jewels are baubles ; 'tis a sin
> To care for such unfruitful things
> One good-sized diamond in a pin,
> Some, not so large, in rings,
> A ruby and a pearl or so,
> Will do for me—I laugh at show."

And if one must needs be serious, what can one want better than " The Anatomist's Hymn" (or " The Living Temple," as the Autocrat prefers to call it), or the beautiful " Hymn of Trust" !

But the great charm of his poetry is the youthful spirit that runs through it. Dr. Holmes declines to grow old ; years won't do it. " You know well enough what I mean by youth and age," he says ; " something in the soul, which has no more to do with the colour of the hair than the vein of gold in a rock has to do with the grass a thousand feet above it." And though he has passed the threescore and ten years that are assigned as the limit of human life, we can reasonably quote his own words about him, and say to all who remark on his age—

> " Call him not old whose visionary hair
> Holds o'er the past its undivided reign ;
> For him in vain the envious seasons roll
> Who bears eternal summer in his soul.
> If yet the minstrel's song, the poet's lay,
> Spring with her birds, or children with their play,
> Or maiden's smile, or heavenly dream of art,
> Stir the few life-drops creeping round his heart ;
> Turn to the record where his years are told ;
> Count his grey hairs—they cannot make him old !"

